# Cryptococcosis mimicking cutaneous cellulitis in a patient suffering from rheumatoid arthritis: a case report

**DOI:** 10.1186/1471-2334-10-239

**Published:** 2010-08-11

**Authors:** Corina Probst, Georg Pongratz, Silvia Capellino, Rolf M Szeimies, Jürgen Schölmerich, Martin Fleck, Bernd Salzberger, Boris Ehrenstein

**Affiliations:** 1 Department of Internal Medicine I, University of Regensburg, Regensburg, Germany; 2 Department of Dermatology and Allergology, Klinikum Vest, Recklinghausen, Germany

## Abstract

**Background:**

*Cryptococcus neoformans *is an encapsulated yeast and the most frequent cryptococcal species found in humans. Cryptococcosis is considered an opportunistic infection as it affects mainly immunosuppressed individuals. In humans, *C. neoformans *causes three types of infections: pulmonary cryptococcosis, cryptococcal meningitis and wound or cutaneous cryptococcosis.

**Case Presentation:**

An 81-year-old woman developed severe necrotizing cellulitis on her left arm without any preceding injury. The patient had been treated with systemic corticosteroids over twenty years for rheumatoid arthritis (RA). Skin biopsies of the wound area were initially interpreted as cutaneous vasculitis of unknown etiology. However, periodic acid Schiff staining and smear analysis later revealed structures consistent with *Cryptococcus neoformans*, and the infection was subsequently confirmed by culture. After the initiation of therapy with fluconazole 400 mg per day the general condition and the skin ulcers improved rapidly and the patient was discharged to a rehabilitation facility. Subsequently surgical debridement and skin grafting were performed.

**Conclusions:**

Opportunistic infections such as cryptococcosis can clinically and histologically mimic cutaneous vasculitis and have to be investigated rigorously as a differential diagnosis in immunosuppressed patients.

## Background

*Cryptococcus neoformans *is an opportunistic encapsulated yeast and the most frequent cryptococcal species found in humans. *Cryptococcus neoformans *is composed of three variants: *C. neoformans *var. *gattii*, var. *grubii*, and var. *neoformans*. *Cryptococcus neoformans *var. *gattii *is encountered mostly in tropical areas, but *C. neoformans *var. *gattii *has been found recently responsible for an ongoing outbreak of cryptococcosis in immunocompetent humans and animals on Vancouver Island, Canada, and surrounding areas [1,2]. *Cryptococcus neoformans *var. *gattii *has now been shown to be different enough from other subspecies for authors suggesting to elevate it to its own species level [3,4].

Infections caused by *C. neoformans *var. *gattii *occur predominantly in apparently healthy hosts [5]. *Cryptococcus neoformans *var. *grubii *and var. *neoformans *have a worldwide distribution. Although, *Cryptococcus neoformans *being isolated from decaying wood, fruits, vegetables, hay and dust [6], bird droppings, especially of pigeons, are an important source for cryptococcal infections [7]. Cryptococcosis is considered an opportunistic infection as it affects mainly immunosuppressed individuals [5,8], for instance of. HIV-infected patients [9], patients with prolonged glucocorticoid-treatment [10,11] after organ transplantation [12,13] or patients with malignancies [14]. However, there are also some reports of cryptococcosis in immunocompetent individuals [15,16]. Cryptococcosis is believed to be acquired by inhalation of the infectious propagule from the environment. In humans, *C. neoformans *causes three types of infections: pulmonary cryptococcosis, cryptococcal meningitis and wound or cutaneous cryptococcosis [17]. The skin lesions typically appear as pedunculated, dome-shaped papules with an umbilicated center. Approximately 15% of patients with systemic dissemination show secondary involvement of the skin [18]. Skin lesions are found in 5% of patients with cryptococcal meningitis [19], and the frequency is higher in liver transplant recipients receiving tacrolimus or in patients infected with serotype D [20]. Primary cutaneous cryptococcosis (PCC) without systemic infection has been perceived to be a rare distinct clinical entity [18].

Here we report the case of a female elderly patient suffering from rheumatoid arthritis, where a cryptococcal cellulitis was initially clinically and histopathologically erroneously diagnosed as a cutaneous vasculitis.

## Case presentation

An 81-year-old woman presented with skin ulceration, swelling, erythema and severe pain of her left arm. The skin lesions developed some months earlier and progressed slowly. The patient reported no accidental injury and no fever. The patient lived in a rural area, had no outdoor hobbies and could not remember any insect bites at presentation. The initial lesion was only a point-shaped lesion. In the course, the primary lesion enlarged and developed into an ulcerous lesion. Concomitantly, the patient suffered from rheumatoid arthritis (RA) and had been treated with on average 20 mg prednisolone daily for over twenty years by her primary care physician. She had never received any disease modifying anti-rheumatic drug (DMARD) therapy. She had no known allergies. She had not travelled for many years and had no close contact to any animals.

**Figure 1:**
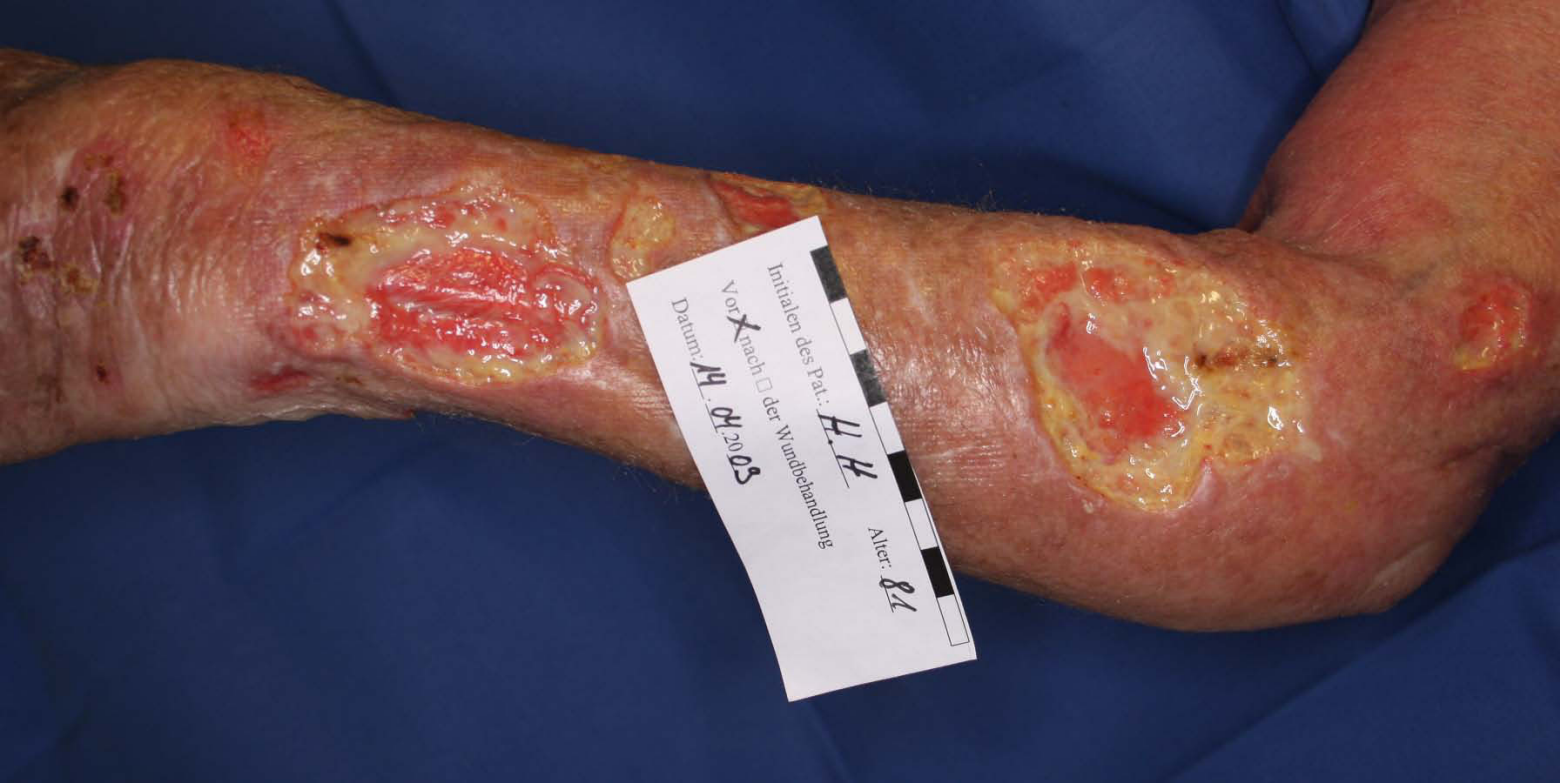
Severe cellulitis on the forearm.

**Figure 2:**
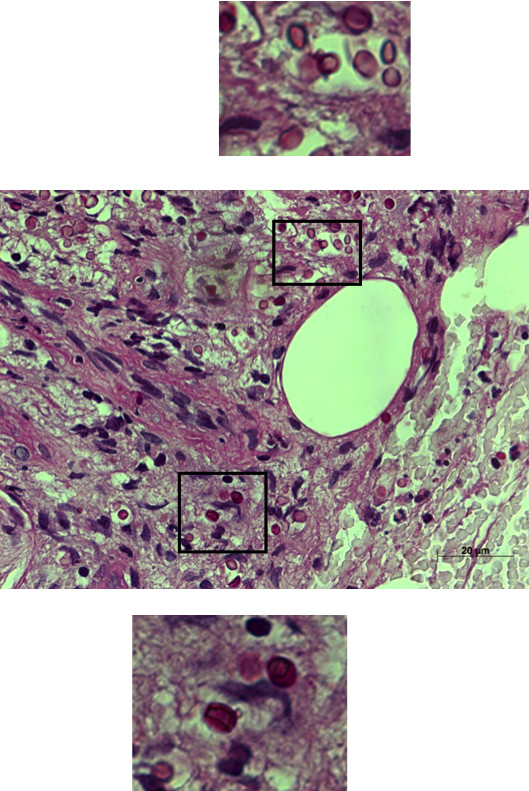
**Cutaneous biopsy specimen**. Periodic acid-Schiff staining with the presence of *Cryptococcus neoformans *(PAS-positive spherules with prominent capsules in a zone of clearance or "halo" around the cells, original magnification × 630).

On examination, the left fore and upper arm exhibited several necrotic deep ulcerations, each approximately 6 cm in diameter. The lesions extended to the muscles and tendons (Figure 1). Regional lymphadenopathy involving the left axillary lymphnodes and cervical lymphnodes were present. The right leg showed the clinical signs of cellulitis. Inflammatory markers were elevated (CRP 280 mg/l, leucocytes 16/nl) and the differential blood count demonstrated an increased percentage of neutrophils. Biopsies and aspirates from several sites of the wound area and blood cultures were obtained and laboratory analyses including immunological parameters were performed. Intravenous piperacillin 3 × 4 g/d combined with sulbactam 3 × 1 g/d was initiated as empirical treatment of bacterial cellulitis and penicillin G 10 Mio. IE was added for presumed erysipelas of the right leg. A rapid improvement of the erysipelas was noted, but no change of the ulcerative cellulitis of the left arm could be perceived. Three pairs of blood cultures remained negative even after prolonged incubation for seven days. Rheumatoid factor (RF), anti-citrullinated peptide antibodies (ACPA), anti-nuclear antibodies (ANA), and anti-neutrophil cytoplasmic antibodies (ANCA) were all tested negative. Chest x-ray and abdominal ultrasound did not reveal any infectious focus. Histological analysis of skin biopsies (haematoxylin-eosin staining) displayed pyoderma. In addition isolated pale spherical structures could be identified, that were also detected by periodic acid-Schiff staining and initially believed to be most likely exogenous contaminations. A second biopsy showed signs of a cutaneous vasculitis consistent with cutaneous involvement of RA. Due to the assumed rheumatoid vasculitis (RV), our colleagues from the Department of Dermatology initiated an empirical treatment with systemic prednisolone. However, there was neither response to the antibacterial therapy nor to the systemic prednisolone in the clinical course. Finally, four weeks after admission, investigations on a third skin biopsy including periodic acid-Schiff (Figure 2) and Gomori's methenamine silver stain revealed *C. neoformans*. This finding was subsequently verified by microbial culture. A wound aspirate from the region of the left elbow was also positive for *C. neoformans*. Retrospectively, one could identify *C. neoformans *as pale spherules in the first biopsy (haematoxylin-eosin staining). Because of these findings, the patient's serum was once tested for cryptococcal antigen with a negative result. A computed tomography (CT) of the lung revealed a peripheral round solid lesion approximately 1 cm in diameter of unknown etiology. Sputum cultures could not be performed because the patient had no cough. The radiologists judged the lung lesion unlikely to represent a cryptococcoma, but the written report did not specifically exclude this diagnosis from the differential diagnosis. The lesion was too peripheral for bronchoscopic guided biopsy and due to the general condition of the patient an open lung biopsy was not performed instead a follow-up CT scan in 3 month was recommended.

Antimycotic therapy with intravenous fluconazole (400 mg daily) was initiated and the empirical intravenous antibiotic therapy deescalated to ceftriaxone. The debris at the base of the ulcera cleared rapidly and after 10 days new formation of granulation tissue was noted on the base of all skin ulcers. The antifungal treatment with daily 400 mg fluconazole was continued orally for further 4 weeks. A follow-up CT scan of the lung after 6 weeks did not reveal any change of the pulmonary lesion. The dose of prednisolone was slowly tapered without a flare of the RA. Three month after the initial admittance and after 6 weeks of rehabilitation, surgical debridement of the necrotic area was performed, and the skin defect was successfully closed with a mesh graft.

## Discussion

As demonstrated by our case report, cutaneous cryptococcal infection can mimic and therefore needs to thoroughly differentiate from rheumatoid vasculitis, which also can manifest with ulcerative skin lesions. Rheumatoid vasculitis typically occurs in patients with long-standing, joint-destructive RA. In one study, the mean duration between the diagnosis of RA and the onset of vasculitis symptoms was 13.6 years [21]. The classic skin lesions of rheumatoid vasculitis are deep cutaneous ulcers on the lower extremities and must be distinguished from pyoderma gangrenosum, which is much less common in RA. In hindsight, the diagnosis of rheumatoid vasculitis appeared unlikely in our patient due to the absence of rheumatoid factor, which is usually highly elevated in patients with RV [22,23]. High titers of rheumatoid factor are reported consistently to be the strongest predictor of the development of rheumatoid vasculitis. Rheumatoid vasculitis in a patient with seronegative RA is very rare manifestation and only a paucity of such case reports have been published to our knowledge [24,25].

*Cryptococcus neoformans *has been recognized to cause systemic infection in immunocompromised patients for a long time. However, the existence of primary cutaneous cryptococcosis a cryptococcal infection of the skin without prior dissemination, as a distinct entity has been discussed only during the last decades [18]. Neuville et al. [18] have proposed criteria to distinguish primary and secondary cutaneous cryptococcosis. Criteria for the diagnosis evidence of primary cutaneous cryptococcosis included the absence of signs of disseminated infection and predominantly a solitary skin lesion on unclothed areas of the skin presenting as a whitlow or phlegmon. In addition, patients may present with regional lymphadenopathy. In our case, there was no solitary skin lesion presenting as a withlow or phlegmon, and no history of a skin injury, no participation in outdoor activities and no exposure to bird droppings could be inquired from the patient. On the basis of these data, the disease of our patient should be classified as anatomically limited manifestations of a disseminated disease state and therefore does not represent primary cutaneous cryptococcosis.

The current guidelines of the Infectious Diseases Society of America (IDSA) recommend amphotericin B based combination therapy with flucytocine as primary induction therapy for all severe forms of disseminated cryptococcal infections (cryptococcosis) followed by fluconazole consolidation therapy [26]. For patients with limited pulmonary cryptococcosis and non-pumonary cryptococcal infections in immunocompetent patients without CNS-involvement initial therapywith fluconazole (400 mg per day) could be considered. Due to the comorbidities and the age of our 81-year-old patient and that there was no evidence for an involvement of the central nervous system or internal organs other than the potentially limited pulmonary involvement, we refrained from administering the standard therapy of amphotericin B and flucytosine for disseminated cryptococcosis [26]. Fortunately, we saw a rapid clinical response to fluconazole in our patient.

## Conclusions

In summary, this case report demonstrates that opportunistic infections, such as cutaneous cryptococcosis, can clinically and histologically mimic cutaneous vasculitis. Rheumatoid vasculitis is exceptionally rare in patients with absent rheumatoid factor. Therefore, cutaneous cryptococcosis and other opportunistic infections have to be rigorously investigated as a differential diagnosis in patients with rheumatoid factor negative RA with cutaneous lesions.

## Competing interests

The authors declare that they have no competing interests.

## Authors' contributions

CP, JS, MF, BS and BE provided the clinical care of the reported patient and all were involved to draft the manuscript. GP, RS, SC were involved in establishing the histopathologic diagnosis and provided the corresponding Figures. All authors read and approved the final version of the manuscript.

## Consent

Written informed consent was obtained from the patient for publication of this case report and any accompanying images. A copy of the written consent is available for review by the Editor-in-Chief of this journal.

## Funding

The funding for this case report was provided by intern resource from our department.

## Pre-publication history

The pre-publication history for this paper can be accessed here:

http://www.biomedcentral.com/1471-2334/10/239/prepub
